# Phytoremediation of potentially toxic elements in a polluted industrial soil using Poinsettia

**DOI:** 10.1007/s12298-021-00980-w

**Published:** 2021-03-27

**Authors:** Fangmeng Xiao, Zhanying Gu, Arbi Sarkissian, Yaxin Ji, RuonanYang, Ling Yang, Qingyang Zeng, Peng Huang, Hanyue Chen

**Affiliations:** 1 Central South University of Forestry and Technology, Changsha, China; 2 Bangor University, Bangor, UK

**Keywords:** Phytoremediation, Phytoextraction, Potentially toxic elements, Ornamental plants, Poinsettia

## Abstract

Potentially toxic elements (PTEs) pollution has become a serious environmental threat, particularly in developing countries such as China. In response, there is a growing interest in phytoremediation studies to identify plant species as designated hyperaccumulators of PTEs in polluted soils. Poinsettia was selected as a candidate species for phytoremediation of six PTEs (Zn, Pb, Hg, Cr, As, Cu) in this study. A pot cultivation experiment (randomized incomplete block experimental design with 5 treatments and 4 blocks) was conducted using contaminated soils gathered from an industrial area in southcentral China. The bioaccumulation factor (BAF), translocation factor (TF), and bioconcentration factor were analyzed to determine the phytoremediation potential of poinsettia potted in different ratios of polluted soils. One-way ANOVA with post-hoc Tukey’s test showed that poinsettia had significant uptake of Zn, Pb, Cu (BAF < 1 and TF < 1, *p* < 0.05) and Hg (BAF < 1 and TF > 1, *p* < 0.05). Poinsettias can therefore effectively accumulate Zn, Pb, and Cu in their lateral roots while extracting and transferring Hg into their leaves. Moreover, poinsettia exhibited tolerance towards As and Cr. Interestingly, it was also observed that PTEs can inhibit the height of potted poinsettia at a certain concentration.

## Introduction

The term "potentially toxic elements" (PTEs), more commonly known as "heavy metals", is considered more appropriate than "toxic" or "heavy metal" as a grouping name for metal(loid)s related to pollution and potential toxicity (Duffus 2002; Shaheen et al. 2013; Pourret and Bollinger 2018). The main reason is that heavy metal, defined as elements with a density greater than 7 g cm^−3^ (other authors choose different limits), does not have biological significance, chemical basis, and definition of authority (Smith 1996; Duffus 2002; Hodson 2004; Madrid 2010; Chapman 2012). In contrast, "Trace elements" (TEs) are associated with their abundance and include metals, metalloids, non-metals, and other elements in soil–plant-animal systems (Shaheen et al. 2013). High concentrations of some TEs like Zn, Pb, Cu, Cr, As, Cd, and Ni are potentially toxic to organisms and may pose long-term risks to ecosystems by circulating in the food web (Arif et al. 2016; Antoniadis et al. 2019).

PTEs pollution has become a pervasive problem globally that poses severe threats to humans and the environment (Cojocaru et al. 2016; Sarwar et al. 2017). The main anthropogenic causes of PTEs pollution are discharged waste from industry and mining, the overuse of agricultural chemicals, sewage sludge used in irrigation, and improper treatment of waste (Odukoya et al. 2018; Yang et al. 2018). In China, the concentrations of PTEs in soils and sediments around factories were estimated to be as high as 36.3%, which is well above the national standard (China 2018). Excessive quantities of PTEs through runoff decrease the quality and productivity of soils and lead to their accumulation in crops, potentially endangering human health (Yang et al. 2018; Khan et al. 2019). For example, excessive Zn intake can lead to depression, prostate cancer, and other chronic diseases (Li et al. 2019). Pb is an extremely toxic metal which have been reported as adversely affecting humans' nervous, endocrine, immune, and circulatory systems (Zhang et al. 2012; Chen et al. 2016). Moreover, As and Cr have been increasingly found in food crops, which poses a severe carcinogenic risk for China's people (Clemens and Ma 2016; Wang et al. 2018). Given the health and environmental risks of toxic metal(loid)s pollution, it is crucial to identify effective strategies for remediating PTEs- contaminated soils.

Phytoremediation of PTEs contaminated soils is an environmentally-sound technique that is more efficient and cost-effective than traditional physical and chemical techniques (John et al. 2009; Bissonnette et al. 2010; Dixit et al. 2015; Sarwar et al. 2017). Phytostabilization and phytoextraction are two commercially promising sub-phytotechnologies of phytoremediation (Raskin 1995; Salt et al. 1995). Phytostabilization uses plants with dense root systems and vegetation cover to stabilize PTEs in the root zone (Salt et al. 1995; Singh 2012; Antoniadis et al. 2017). Phytoextraction, the most common phytotechnology employed, uses plants to absorb various PTEs from the soil and translocate them to their aerial organs (Raskin 1995; Peng et al. 2009; Antoniadis et al. 2017). Some plants that can tolerate and translocate high PTEs in their aerial organs without toxic symptoms are termed hyperaccumulators (Brooks 1977; Baker and Brooks 1989; Memon and Schroder 2009). Conversely, non-hyperaccumulators (or simply 'accumulators') store PTEs in their roots rather than translocating them to aboveground parts. Investigating the phytoremediation potential of ornamental plants that can be sold commercially can incentivize their use in remediating soils given the added profitability (Schwitzguébel 2015).

Some studies have found various native plants with hyperaccumulation tendencies of PTEs occurring naturally around metallurgy and mining factories (Čudić et al. 2016; Sasmaz et al. 2016). However, these plants are mostly herbaceous annuals with little commercial value, or that cannot be self-sustaining (Moreno-Jiménez et al. 2011). Moreover, since large-scale planting requires substantial initial investments, interest has shifted towards identifying ornamental species with economic benefits or by-product generation to sustain phytoremediation efforts (Chintakovid et al. 2008; Chaturvedi et al. 2014; Nakbanpote et al. 2016). The ornamental plant *Euphorbia milli* has been shown to effectively remediate moderately-contaminated soils of Cr (Ramana et al. 2015).

Poinsettia (*Euphorbia pulcherrima* Willd. et Kl.), a perennial shrub of the spurge family (*Euphorbiaceae*), is a commercially important pot and cut flower species cultivated globally, especially as a Christmas ornamental plant (Mabberley 1997; Allaby 2016). Current breeding research of poinsettia has focused on its cultivation and management techniques (Zhou 2009; Meng 2014). Since poinsettias are commercially more popular than *Euphorbia milli*, it is of considerable importance to explore whether poinsettia has the remediation potential of PTEs contaminated soils to promote the wide commercial application of phytoremediation technology. Several studies involving phytoremediation pot experiments have reported that, plants were only exposed to one or two types of PTEs at a time, which may reduce plants to show their true phytoremediation potential due to neglecting the effects of a multi-element environment (Antoniadis et al. 2017). Therefore, in this study, we directly collected the contaminated soil in the industrial area for the pot experiment and studied poinsettia's ability to remediate six common PTEs, including toxic metals (Zn, Pb, Hg, Cr, Cu) and metalloids (As).

Since the large-scale establishment of factories (old industrial areas) began in 1953 in Zhuzhou city of Hunan province in southcentral China, PTEs pollutants have been continually discharged for over 20 years without adequate treatment (Dou et al. 2008). As a result, Zhuzhou has become one of the most PTEs polluted cities in China (Dou et al. 2008). It is of considerable interest to remediate these soils to prevent PTEs from entering the food chain and posing health and environmental risk. Thus, the main aim of this study was to: (1) evaluate the growth of poinsettia in industrial soils contaminated by PTEs; (2) evaluate the hyperaccumulation and phytoremediation potential of poinsettia for Zn, Pb, Hg, Cr, As, Cu.

## Materials and methods

### Study sites and soil collection

Soils were collected from Xintang Village (113° 4′ E, 27° 43′ N) in Zhu Zhou city using the diagonal method and a mixed broad-leaf forest patch in the Central South University of Forestry and Technology in Changsha city (111° 53′ E ~ 114° 15′ E; 27° 51′ N ~ 28° 41′ N), respectively. Topsoil samples (0–20 cm) were taken at diagonal intervals of 4 m along a more than 0.13 ha plot. Then 100 g of soils were obtained by repeatedly using the point-centered quarter method (Dix 1961): All the soils were mixed well, laid out in a square, and divided diagonally into four parts. Two diagonal parts were randomly combined into one part, repeated until a random sample of 100 g was obtained, and then tested their PTEs contents by the flame atomic absorption spectrophotometer (WFX-120B) (Russell et al. 1957). The rest of the soil was used for planting.

### Experimental design

A randomized incomplete block experimental design was used. Based on soil samples' different volume ratios from industrial and non-industrial areas, soil samples were divided into five treatment groups with four replicates in each group. Treatment 1 (control group) consisted of all non-industrial soils, while treatment 5 was 100% industrial. Treatments 2 to 4 were a mixture of industrial and non-industrial soils, with the volume proportions of industrial soils being 25%, 50%, and 75%, respectively. Soil samples were first air-dried and sieved (3 mm), followed by an analysis of the PTEs, physical and chemical properties for each of the five treatment groups (Andreazza et al. 2010). The soil electrical conductivity of each treatment was measured by soil parameter detector (RS-ECTH-SC-1). Each of the five treatment groups was set up into four blocks consisting of 10 plastic pots (19.1 cm diameter) per block, 40 pots in total. Pots were then packed with soil mixtures in the ratios described (2 pots per treatment per block). Each pot was later planted with two poinsettia cuttings (80 cuttings in total).

### Plant growth and phytoaccumulation analysis of PTEs

The pot culture experiment took place from April to December 2019. To ensure that the pre-experimental plant material was free of PTEs and genetically consistent plant material was obtained, the rooted cuttings of poinsettia were used for culturing in a clean medium of vermiculite and perlite without pollution then transplanted to the experimental medium according to experimental design (Ramírez et al. 2020). Poinsettia cuttings were inserted into a substrate for rooting (one cutting per substrate) in April. After one month, 80 rooted plants with similar height and basal diameter were selected and transplanted into the plastic pots of different treatments described above, with two plants per pot. Regular watering and weeding were done during plant growth. After 67 days, plant height and stem basal diameter were measured every 14–20 days. Plants were harvested from the pots, and all green and red leaves were picked after six months (in December). Since most of the poinsettias did not defoliate early, the samples of roots, leaves, and stems were all collected simultaneously.

Fresh leaves (red and green) of all plants were weighed immediately after being harvested. After that, all fresh leaves were scanned with the e-Expression 11000XL Root Scanner to the computer and then each red and green leaf area was calculated using the ImageJ 1.50i software. 0.2 g of fresh green and red leaves from each plant were obtained and then measured by a UV1201 spectrophotometer (Mapada-3000 PC) to calculate the chlorophyll and anthocyanins content. All the measurements were repeated twice.

Before oven-drying, the leaves for 72 h at 65 °C and weighting, all remaining red and green leaves from the same treatment group in four blocks were mixed separately, from which two red leaves and two green leaves were randomly selected for detection of PTEs content with the flame atomic absorption spectrophotometer (WFX-120B) (Russell et al. 1957). Measurement of PTEs in roots and stems was consistent with leaves, whereas the post-test soil required the repeated point-centered quarter method selection of 100 g of sample for PTEs measurement. In particular, the whole root system was obtained as much as possible since many TEs tend to accumulate in large quantities in the fine roots (Vamerali et al. 2009).

The PTEs accumulation potential of the poinsettia was evaluated by determining bioconcentration factor (BCF), bioaccumulation factor (BAF), and translocation factor (TF). Values of BCF higher than 1 indicate that the plant is likely a hyperaccumulator (Usman et al. 2019). Values of BAF and TF are used to evaluate plant phytoremediation potential (Bhatti et al. 2018). BAF higher than 1 indicates that the plant has the ability of phytostabilization, and a TF higher than 1 means that the plant can absorb and remove PTEs into aerial organs for phytoextraction:

$$\begin{array}{l} {\rm{BCF }} = {{\rm{C}}_{{\rm{root}}}}/{{\rm{C}}_{{\rm{soil}}}}\\ {\rm{BAF}} = {{\rm{C}}_{{\rm{shoot}}}}/{{\rm{C}}_{{\rm{soil}}}}\\ {\rm{TF }} = {{\rm{C}}_{{\rm{shoot}}}}/{{\rm{C}}_{{\rm{root}}}} \end{array}$$

where C_root_, C_shoot_, and C_soil_ are metal concentrations (μg g^−1^) in the root, shoot, and soil, respectively.

### Statistical analyses

In order to evaluate statistically significant differences among values, all data were analyzed using SPSS 18.0 software. Wet and dry weight measurements, chlorophyll and anthocyanin content, as well as PTEs content of plant tissues (taproot, lateral root, stem, and leaf), were analyzed using one-way ANOVA and post-hoc Tukey's test (*p* < 0.05). A two-way repeated-measures ANOVA was used to analyze the relationship between plant height and stem diameter growth, treatment, and time.

## Results

### Soil analysis

The soil in both the experimental and control groups was red loam. The physical and chemical soil analysis (Table 1) showed that the forest soil was weakly acidic (pH = 6.84), while the contaminated soil approached neutrality. The pH and EC values of polluted soil in Zhuzhou are higher than those of the control group, which may be because the sampling site of polluted soil in Zhuzhou used to be agricultural land, and the high amount of chemical fertilizer used led to the higher content of water-soluble ions in the soil. The total organic carbon (TOC), total nitrogen (Total N), and available phosphorus (Olsen-P) decreased as the proportion of industrial soil increased (*p* < 0.05). Moreover, all experimental groups exceeded the control value except for 25% and 50% treatment groups of Cr (*p* < 0.05) according to the screening and control values for PTEs in agricultural land in China (China 2018).

**Table 1:** Physico-chemical characteristics of soils in each treatment before the experiment

Treatments	pH	EC (us cm^−1^)	TOC (g kg^−1^)	Total N (g kg^−1^)	Olsen-P (mg kg^−1^)	Zn (µg g^−1^d. wt)	Cu (µg g^−1^d. wt)	Pb (µg g^−1^d. wt)	Cr (µg g^−1^d. wt)	As (µg g^−1^d. wt)	Hg (µg g^−1^d. wt)
0%	6.84 ± 0.00e	115 ± 0.00e	33.6 ± 0.00a	2.47 ± 0.00a	16.6 ± 0.00a	1006 ± 0.06e	251 ± 0.50e	5.12 ± 0.01e	877 ± 1.34e	146 ± 0.02a	4.31 ± 0.15c
25%	6.87 ± 0.00d	135 ± 0.00d	29.0 ± 0.00b	2.34 ± 0.00b	16.3 ± 0.00b	1152 ± 0.02d	284 ± 0.01d	127 ± 0.01d	933 ± 0.01d	145 ± 0.01b	4.78 ± 0.09c
50%	6.91 ± 0.00c	170 ± 0.00c	24.3 ± 0.00c	2.21 ± 0.00c	16.1 ± 0.00c	1298 ± 0.01c	316 ± 0.60c	249 ± 0.02c	975 ± 00.01c	144 ± 0.02c	5.51 ± 0.05b
75%	6.94 ± 0.00b	204 ± 0.00b	19.6 ± 0.00d	2.09 ± 0.00d	15.8 ± 0.00d	1444 ± 0.02b	351 ± 0.01b	371 ± 0.01b	1017 ± 0.20b	143 ± 0.02d	6.14 ± 0.09a
100%	6.98 ± 0.00a	235 ± 0.00a	14.9 ± 0.00e	1.96 ± 0.00e	15.6 ± 0.00e	1590 ± 0.03a	385 ± 0.01a	493 ± 0.01a	1060 ± 0.04a	142 ± 0.02e	6.33 ± 0.15a
CV (%)	57.7	75.8	79.6	64.8	60.1	45.6	46.4	1.20	53.5	59.2	45.8

*CV* means the coefficient of variation of the means (The same as below). *ES* means electrical conductivity, *TOC* means total organic carbon, and *d.wt* means dry weight. Data are shown as mean values ± standard error. Different letters in the column represent significant differences between treatments (*p* < 0.05) using Tukey's test

### Plant growth under different treatments

**Fig. 1:**
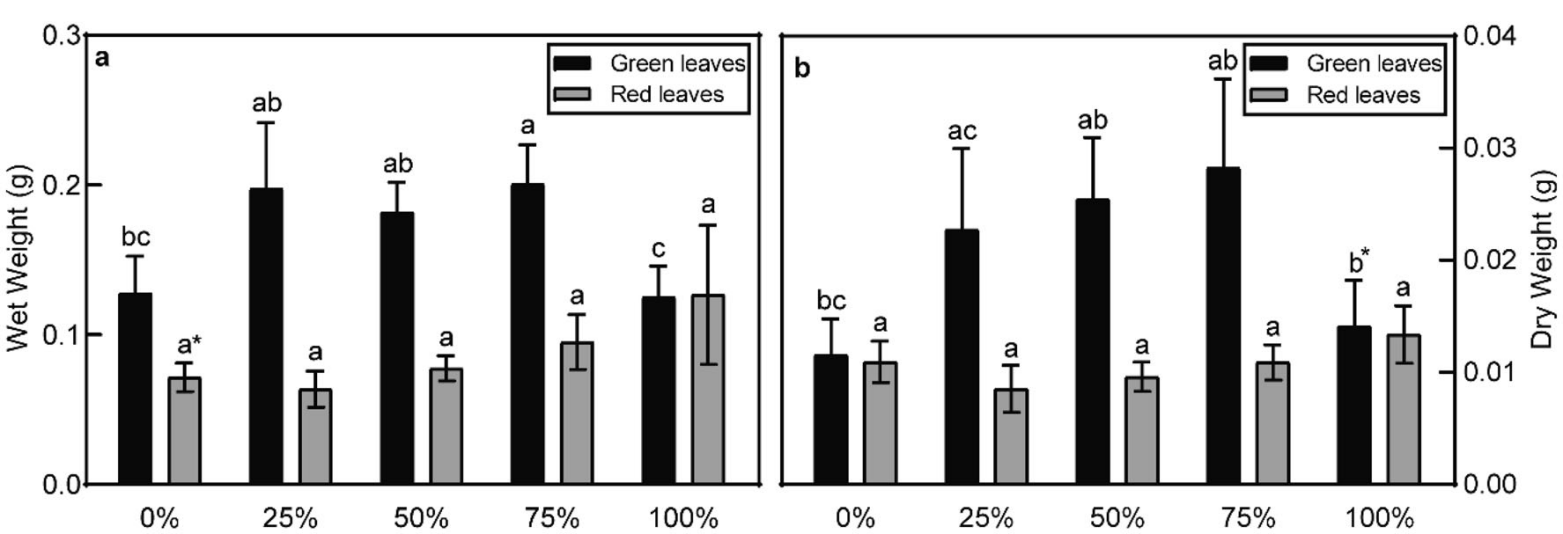
Wet weight (**a**) and dry weight (**b**) of the green leaves and red leaves of poinsettia plants after growing in industrial and non-industrial soils with different volume ratios: non-industrial soil (control group), 25% contaminated soil, 50% contaminated soil, 75% contaminated soil and 100% contaminated soil. *Different letters in the same color bar represent significant differences (*p* < 0.05) with the Tukey test. Error bars were calculations of standard error

Figure 1 showed that there was no significant difference in the dry and wet weights of both green and red leaves between the experimental treatments and control group (0% treatment group) (*p* > 0.05) except the wet weight of green leaves in 75% treatment group (0.05 g per leaf higher than 100% treatment group, *p* < 0.05). The wet weight of green leaves in the 100% treatment group (0.1 g per leaf) was significantly lower compared to that of the 25% treatment group (0.18 g per leaf), 50% treatment group (0.17 g per leaf), and 75% treatment group (0.18 g per leaf) (*p* < 0.05) (Fig. 1a). In addition, the dry weight of the 25% treatment group was 0.13 g per leaf higher compared to the 100% treatment (*p* < 0.05) (Fig. 1b).

**Fig. 2:**
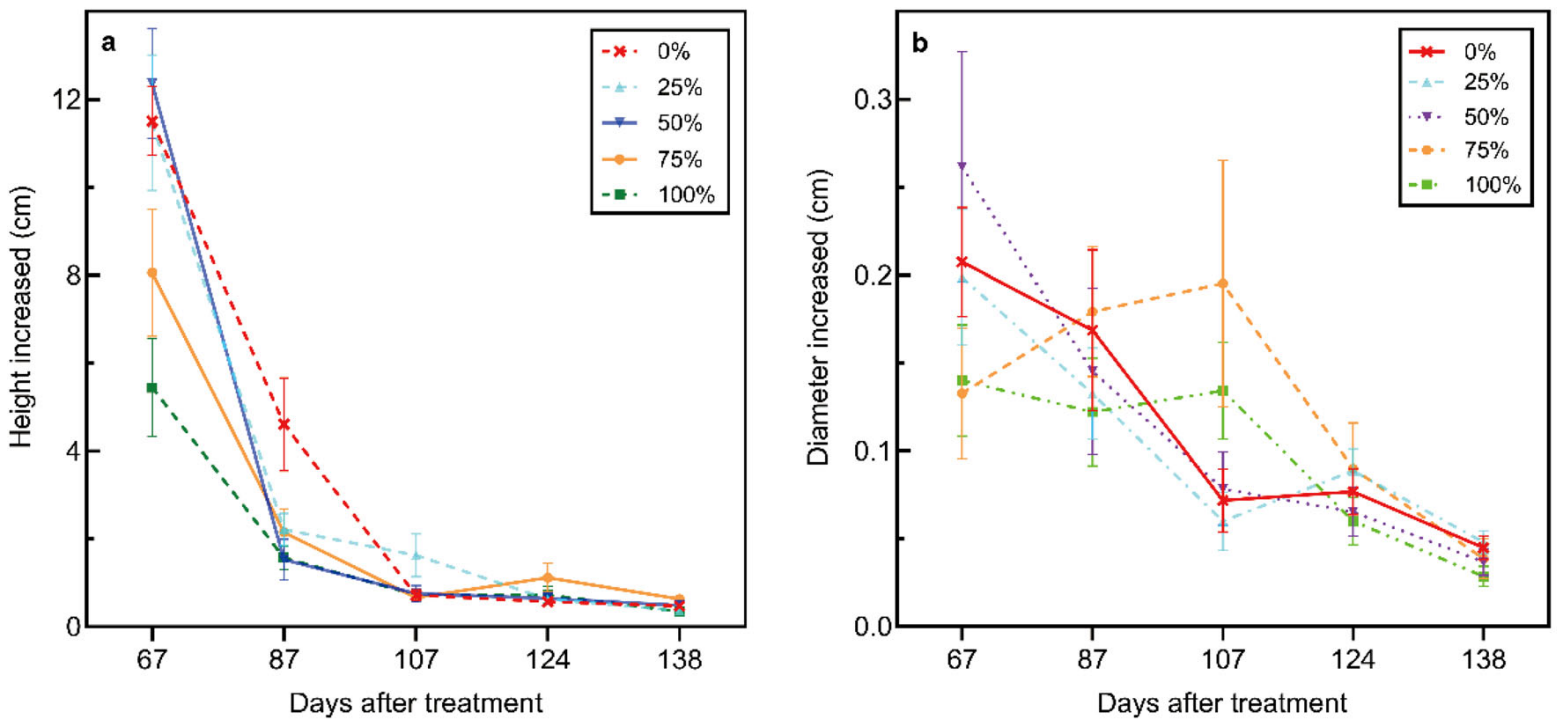
Changes in the height and diameter of poinsettia grown in different contaminated soils

The increase in height of the control group and 50% treatment group was significantly higher compared to that of the 100% treatment group after 67 days of growth in pots (*p* < 0.001) (Fig. 2a). In five treatment groups, the first increase in height (on the 67th day) was significantly different from the other four subsequent times (*p* < 0.001). In 25% treatment group, the increased height in day 67th-87th was higher compared to that in day 107th-124th (1.6 cm) and day 124th-138th (1.9 cm) (*p* < 0.05), while in 100% treatment group, it was higher compared to that in day 87th-107th (0.9 cm) and day 124th-138th (1.2 cm) (*p* < 0.05). The impact of treatment on the increase of diameter was not statistically significant (*p* > 0.05) (Fig. 2b).

### Changes in chlorophyll and anthocyanin content and leaf area

**Fig. 3:**
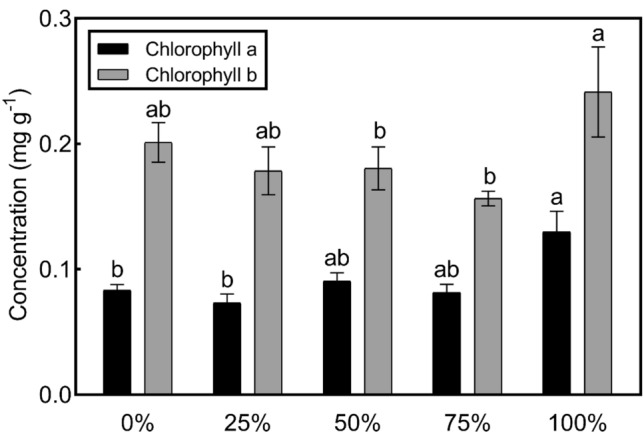
Comparison of poinsettia chlorophyll a and chlorophyll b contents under different treatments. *Different letters in the same color bar represent significant differences (*p* < 0.05) with the Tukey test. Error bars were calculations of standard error

Among the green leaves of 100% treatment group, the average concentration of chlorophyll a (0.13 ± 0.06 mg g^−1^) was 0.05 mg higher compared to the control group (0.08 ± 0.16 mg g^−1^) (*p* = 0.007) and 0.06 mg higher compared to the 25% treatment group (0.07 ± 0.03 mg g^−1^) (*p* = 0.000). For chlorophyll b, the average concentration of the 100% treatment group (0.13 ± 0.06 mg g^−1^) was 0.06 mg higher compared to the 50% treatment group (0.08 ± 0.16 mg g^−1^) (*p* = 0.020) and 75% treatment group (0.07 ± 0.03 mg g^−1^) (*p* = 0.020). The pairwise comparison between the other groups has no statistically significant difference (*p* > 0.05) (Fig. 3).

**Fig. 4:**
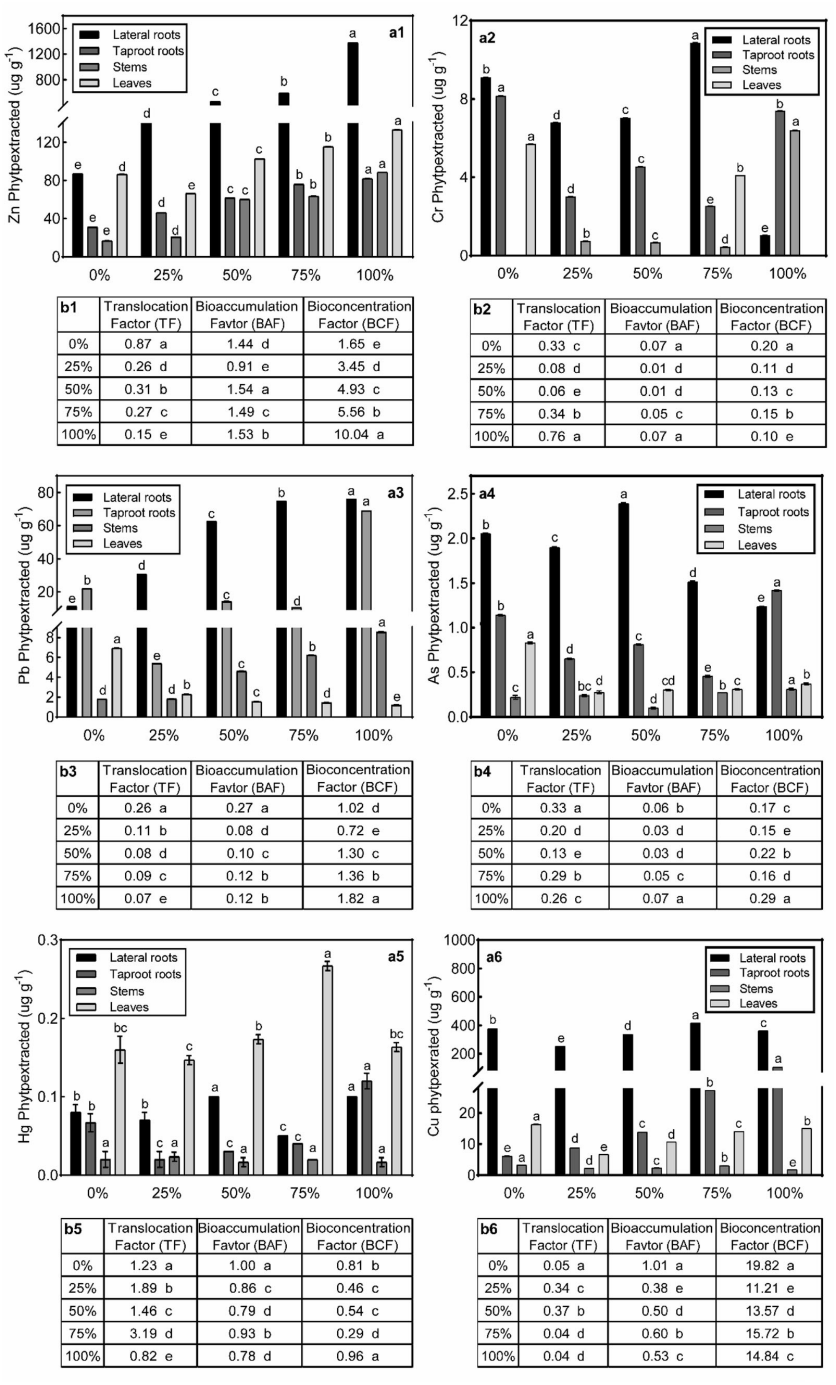
**a** Zn, Pb, Hg, Cr, As, Cu concentration in roots, stem, and leaves after growing in industrial and non-industrial soils with different volume ratios: native soil (*control*), 25% contaminated soil, 50% contaminated soil, 75% contaminated soil and 100% contaminated soil. **b** The PTEs translocation factor (TF), bioaccumulation factor (BAF), and bioconcentration factor (BCF). *Different letters in the same color bar and column represented significant differences (*p* < 0.05) with the Tukey test. Error bars were calculations of standard error

Although the average concentration of chlorophyll (leaf area) in the 100% treatment group was significantly higher (lower) compared to the 25%, 50%, and 75% treatment group (*p* < 0.05), there was no significant difference between them in 100% treatment group and control group soil (Table 2). The mean green leaf area of all treatments ranged from 4.6 cm^2^ to 9.4 cm^2^, while red leaves ranged from 2.6 cm^2^ to 3.1 cm^2^. There was no significant difference in anthocyanin content and red leaf area size between groups (*p* > 0.05).

**Table 2:** Comparison of the content of chlorophyll and leaf area in green poinsettia leaves and the content of anthocyanins and leaf area in red leaves

Treatment	Green leaves		Red leaves	
	Chlorophyll (mg g^−1^)	Leaf area (cm^2^)	Anthocyanin (nmol g^−1^)	Leaf area (cm^2^)
0%	0.28 ± 0.07ab	7.1 ± 6.10ab	1157 ± 798a	2.7 ± 2.90a
25%	0.23 ± 0.06b	9.4 ± 8.81a	1131 ± 620a	2.6 ± 2.81a
50%	0.25 ± 0.04b	8.2 ± 6.03a	1150 ± 618a	2.8 ± 2.59a
75%	0.24 ± 0.04b	8.8 ± 5.12a	1021 ± 558a	3.1 ± 3.22a
100%	0.37 ± 0.17a	4.6 ± 4.17b	1177 ± 526a	2.8 ± 2.90a

Values were shown as mean ± standard error. Different letters in the column represented significant differences (*p* < 0.05) with the Tukey test

### Phytoextraction and Phytostabilization of PTEs

Zn absorption increased significantly with the increase of the PTEs content in the treatment group both in the whole plant and in both aerial and belowground organs of the plant (*p* < 0.05) (Table 3, Fig. 4a1). However, the content of Zn absorbed in the lateral roots of the poinsettia was significantly higher compared to that in other parts of poinsettia (*p* < 0.05). The BCF of poinsettia for Zn increased from 1.65 (0% treatment) to 10.04 (100% treatment), and BAF was more than 1 except for the 25% treatment group. However, TF decreased from 0.87 (0%) to 0.15 (100%) (Fig. 4b1).

**Table 3:** Content of PTEs in dried soil and the whole plant after harvesting plants from the different volume of contaminated soils

	Zn (µg g^−1^d. wt)		Cu (µg g^−1^d. wt)		Pb (µg g^−1^d. wt)	
	Soil	Plant	Soil	Plant	Soil	Plant
0%	72 ± 0.01e	221 ± 0.86e	19 ± 0.01e	403 ± 0.04c	33 ± 0.01e	42 ± 0.01d
25%	95 ± 0.01d	416 ± 0.15d	23 ± 0.02d	271 ± 0.15e	51 ± 0.02d	40 ± 0.01e
50%	106 ± 0.02c	684 ± 0.01c	26 ± 0.01c	364 ± 0.01d	59 ± 0.01c	83 ± 0.01c
75%	120 ± 0.01b	847 ± 0.02b	28 ± 0.02b	461 ± 0.02b	63 ± 0.01b	93 ± 0.01b
100%	145 ± 0.02a	1676 ± 0.04a	32 ± 0.01a	486 ± 0.02 a	80 ± 0.01a	155 ± 0.01a
CV (%)	43.7	67.7	47.1	19.8	42.5	52.3

	Cr (µg g^−1^d. wt)		As (µg g^−1^d. wt)		Hg (µg g^−1^d. wt)	
	Soil	Plant	Soil	Plant	Soil	Plant
0%	86 ± 0.01e	23 ± 0.01a	18 ± 0.01a	4.2 ± 0.03a	0.19 ± 0.02d	0.33 ± 0.01c
25%	93 ± 0.01a	11 ± 0.03e	17 ± 0.02b	3.1 ± 0.02d	0.20 ± 0.01cd	0.26 ± 0.00d
50%	92 ± 0.01b	12 ± 0.01d	15 ± 0.01c	3.6 ± 0.00b	0.24 ± 0.01b	0.33 ± 0.00c
75%	92 ± 0.02c	18 ± 0.01b	12 ± 0.02d	2.5 ± 0.01e	0.31 ± 0.01a	0.38 ± 0.01b
100%	88 ± 0.02d	15 ± 0.01c	9 ± 0.01e	3.3 ± 0.01c	0.23 ± 0.01bc	0.40 ± 0.05a
CV (%)	59.9	29.0	78.4	17.4	44.9	15.1

Values are shown as mean ± standard error, and *d.wt* means dry weight. Different letters in the column represent significant differences (*p* < 0.05) with the Tukey test

Pb content in the whole plant reached 155 µg g^−1^ in the 100% treatment group, while it was only 42 µg g^−1^ in the control group (Table 3). With the increase of the proportion of industrial soils, the content of Pb absorbed in the lateral roots (11.30—80.00 µg g^−1^) and the stems (1.83–8.58 µg g^−1^) increased significantly (*p* < 0.05). Conversely, Pb content in leaves decreased from 6.94 µg g^−1^ to 1.21 µg g^−1^ (Fig. 4a3). Pb concentration in taproots of the 100% treatment group (69.1 µg g^−1^) was significantly higher compared to other groups and was close to that in the lateral roots (76.0 µg g^−1^, *p* < 0.05). Both TF and BAF of poinsettia to lead were less than 1. However, the BCF values in each group were more extensive than 1 and reached 1.82 in the 100% treatment group, except 0.72 in the 25% treatment group (Fig. 4b3).

The Hg content of the whole plant increased in consecutive treatment groups (Table 3). The taproots and the lateral roots absorbed most Hg in 50% (0.10 µg g^−1^) and 100% (0.12 µg g^−1^) treatment groups, which were significantly higher than those in the control group (0.08 µg g^−1^) and 25% treatment groups (0.02 µg g^−1^, *p* < 0.05) (Fig. 4b5). The 75% treatment group had the highest Hg content in the leaves, a difference of 0.12 µg g^−1^ from the lowest group (25%). The BAF and BCF values of Hg in industrial soils were all lower but closed to 1. The TF, however, higher than 1 in all groups, except 0.82 in the 100% treatment group (Fig. 4b5).

The whole plant in the experimental group had less Cr compared to control group (Table 3). The lateral roots absorbed significantly less Cr in the 25% treatment group compared to that in the control group (9.09 µg g^−1^) (Fig. 4a2). As the industrial soil ratio increased, the Cr absorption in lateral roots also increased significantly, reaching 10.9 µg g^−1^ in the 75% treatment group (*p* < 0.05), but it was significantly less in the 100% treatment group (2 µg g^−1^, *p* < 0.05). Taproot absorbed the least Cr (2.52 µg g^−1^) in the 75% treatment group and increased to 7.39 µg g^−1^ in the 100% treatment group, but still less compared to the control group (8.16 µg g^−1^, *p* < 0.05). In leaves, Cr was only presented in the control group (5.69 µg g^−1^) and 75% treatment group (4.10 µg g^−1^). The TF and BAF at the control group were 0.33 and 0.07, respectively. From 50 to 100%, TF continued to increase and tended to approach 1 (0.76 in 100% treatment group), while BAF and BCF in all treatment groups were far less than 1 (Fig. 4b2).

The poinsettia took up the most As in the control group (4.2 µg g^−1^) compared to all experimental groups (Table 3). However, plant's absorbed As varied in different organs. Compared to other organs, the lateral roots took up the most As in the 50% treatment group (2.39 µg g^−1^) (Fig. 4a4). In all industrial soil treatment groups, the TF, BAF, and BCF values were far less than 1 (Fig. 4b4).

Plants could store more Cu in 75% (461 µg g^−1^) and 100% (486 µg g^−1^) treatment groups compared to that in the control group (403 µg g^−1^) (Table 3). Cu absorbed by lateral roots was highest in the 75% contaminated soil group (416 µg g^−1^), followed by the control group (377 µg g^−1^) (Fig. 4a6). The content of Cu absorbed by the leaves in the contaminated soil increased in consecutive treatment groups, but all were less compared to the control group (16.3 µg g^−1^). Both the TF and BAF of poinsettia for Cu are lower than 1 in the industrial soils. But the BCF value is particularly high, reaching a maximum of 19.82 in the control group (Fig. 4b6).

## Discussion

Poinsettia is a fast-growing plant that can grow up to 15 m in height, but potted varieties are generally less than 0.3 m (Hu et al. 2003). Therefore, most of the research is focussed on controlling the height of poinsettia plants (Yuan et al. 2004; Zhou 2009). In this study, the PTEs content in the soil did not significantly affect the increase in poinsettia stem diameter but it affects the height of the plant. The results indicated that the higher the industrial soil ratio, the earlier it showed an inhibitory effect on plant height. The increase of plant height in the 100% treatment group began to show significant suppression within 67 days. After 138 days, the plant height was about 20–30 cm, equivalent to the Chinese poinsettia plant height standard (Ran et al. 2016). One reason may be the accumulation of Cr in the stems of plants (TF = 0.76) inhibited the metabolism of shoots (Singh et al. 2013), and adult plants are less sensitive to metal toxicity compared to those in juveniles (Nehnevajova et al. 2012), but it cannot be ruled out that the contaminated soil provides the plant with less organic matter, making the element less available to the plant (Antoniadis et al. 2017). This observation may provide new ideas for plant height control of potted poinsettias and reduce the cost of purchasing growth inhibitors.

In general, higher the dry and fresh weight of leaves (green and red leaves), the more organic matter it accumulates (Gu et al. 2009). In this study, PTEs in industrial soils had no side effect on the dry weight of the red and green leaves of poinsettias. However, when the ratio of industrial soils was 75%, it could increase the wet weight of green leaves. With the increase of PTEs content in the soil, the chlorophyll in the green leaves slightly increased (mainly to promote the synthesis of chlorophyll b), but there were no significant differences in leaf area, indicating that PTEs in industrial soils has no significant effect on the photosynthesis and green leaf growth of poinsettia. Some leaves of poinsettia turn red (bracts) due to the increase in anthocyanins, and the bracts are one of the most ornamental organs of the poinsettia. Results showed that PTEs in industrial soils had no significant effect on the synthesis of anthocyanins and leaf area of the poinsettia. Therefore, poinsettias can grow normally in industrial soils.

There are no established standards for the screening of hyperaccumulator plants but mainly include two points. First, to determine if the PTEs accumulated in the plant is more than 10 times the normal uptake level of the plant (Bi et al. 2006) or exceeds some known threshold (Zn > 10,000 mg · kg^−1^; Pb, Cu, As > 1000 mg · kg^−1^) (Brooks 1977; Chaney et al. 1997; Baker and Brooks 1989). For instance, *Arabidopsis balleri* was a Zn hyperaccumulator that could accumulate 32,000 mg kg^−1^ Zn without showing any phytotoxicity symptoms (Zhao et al. 2000). Following this standard, Poinsettia is not a hyperaccumulator but has excellent potential for hyperaccumulation of Zn because the concentration of Zn in plants increased with the ratio of industrial soil increasing (Table 3). The second method is to compare TF, BAF, and BCF values (Wei and Chen 2001; Zu et al. 2004; Yoon et al. 2006; Cui et al. 2007; Li et al. 2007; Malik et al. 2010; Bedabati Chanu and Gupta 2016; Cojocaru et al. 2016). In this study, poinsettia had a BCF > 1 and TF < 1 when Zn, Pb, and Cu were analyzed, indicating the potential for phytostabilization (Yoon et al. 2006). On the contrary, the poinsettia can transport a high level of Hg to the leaves (BAF < 1 and TF > 1), suggesting its potential for phytoextraction (Were et al. 2017). Interestingly, there are more Cr and As taken up in roots and leaves of the control group compared to 100% treatment group. For Cr, it is essential to clarify that only 75% and 100% of the treatment groups had soil Cr levels above the standard before the experiment. Therefore, poinsettias grown in soil with Cr overload may inhibit Cr uptake in the lateral roots, and the Cr in the leaves may have been transferred to the stems (Singh et al. 2013). However, the TF of Cr tends to approach 1 with the increase of PTEs content in the soil, indicating that poinsettia may have the potential of phytoextraction of Cr. Furthermore, the TF value of Cr in the 100% treatment group of poinsettias (TF = 0.76) was higher compared to the *Euphorbia mill* (TF = 0.73), which was considered to have phytoremediation potential (Ramana et al. ). More particularly, As was more severely exceeded in non-industrial soils compared to that in industrial soils before the experiment. As the soil As cont^2015^ent increased, the As levels in lateral roots and leaves increased while that in primary roots and stems decreased. Nevertheless, poinsettia had no phytoremediation for As because the indices were all far less than 1. Further, field experiments are needed to observe the phytoremediation of poinsettias on PTEs, as phytoremediation can be assisted in pot experiments by amendments of releasing agents, but the remediation factors may be lower under real field conditions (Neugschwandtner et al. 2008; Chen et al. 2016).

The results indicated that the poinsettia concentrated PTEs mainly in the lateral roots. This was consistent with the fact that many ornamental plants accumulated PTEs in their roots (Chintakovid et al. 2008; Trigueros et al. 2012; Cui et al. 2013; Chaturvedi et al. 2014; Pérez-López et al. 2014). Therefore, they can be used to remediate contaminated soil to some extent through phytostabilization while landscaping and selling for income. After harvest, the metal-enriched biomass usually needs to be incinerated after harvesting to recover residual PTEs and avoid secondary contamination from plant litter (Nakbanpote et al. 2016). The poinsettia accumulated Zn, Pb, and Cu in the roots, so the aerial organs of poinsettia can still be harvested and marketed as cut flowers for their ornamental value. Moreover, the aerial organs of the poinsettia did not accumulate large amounts of TEs, reducing the risk of transfer of PTEs to the food chain (Dominguez et al. 2008). Thereby, poinsettia may be an essential candidate for the use of Zn, Pb, Cu phytostabilization, and Hg phytoextraction in industrial soils contaminated with PTEs.

Various metal ions dissociated from their complex forms on the surface of the roots and were stored in large amounts in the root branch exosome (Arif et al. 2016). Trees with phytoremediation capabilities absorb PTEs through their extensive root systems and transport them to aerial organs (Vincent et al. 2018). However, their woody characteristic is not conducive to PTEs accumulation (Ahmad 2016). Among the 54 tree species, the maximum absorption of Cu, Pb, and Zn were only 371, 27, and 539 µg g^−1^ (Shang et al. 2019), all lower compared to the value of poinsettia absorption (486, 155, and 1676 µg g^−1^). Therefore, shrubs may be more efficient at absorbing PTEs compared to trees. As for herbaceous perennials, they need large planting areas and the mature grasses need to be removed each year promptly. Many herbs can accumulate large amounts of PTEs primarily because of their high biomass (Hou et al., 2020). For example, *Pennisetum sp.* has higher biomass compared to both the Cd hyperaccumulator *Sedum Plumizincicola* and the Cu tolerant *Elsholtzia splendens*, so it can accumulate and remove more Cd and Cu (Xu et al. 2019). From these perspectives, ornamental shrubs may be a better substitute for herbs.

## Conclusion

By comparing the growth status of poinsettia in 5 different levels of polluted soils, we deduced the following conclusions. (1) Poinsettia grows well in PTEs contaminated soils, and PTEs (possibly chromium) may be able to control poinsettia plant height. (2) Our study showed poinsettia was not a hyperaccumulator of Zn, Pb, Hg, Cr, As, and Cu. However, poinsettia had the potential of hyperaccumulator for Zn and phytoremediation for Cr, which need to be further studied. (3) Poinsettia characterized high potential for phytoextraction of Hg and phytostabilization of Zn, Pb, and Cu in industrial soils. Besides, it can tolerate As and Cr. In future experiments, methods to improve the TF of Zn, Pb, Hg, and Cu in poinsettia can be studied, such as adding chelators and cooperating with microorganisms to speed up the phytoremediation process. (4) Perennial ornamental shrubs, such as poinsettias, may be more suitable for phytoremediation applications than some trees and herbs.

## Data Availability

All data and materials support the published claims and comply with field standards.

## Code availability

Software application or custom code support the published claims and comply with field standards.
